# Supplementary material for: MXene Core-Shell Nanosheets: Facile Synthesis, Optical Properties, and Versatile Photonics Applications
Source: Nanomaterials (Basel). 2021 Aug 3;11(8):1995. doi: 10.3390/nano11081995 (PMC8398170; doi:10.3390/nano11081995)
Supplement: Supplementary file 1 [file nanomaterials-11-01995-s001.zip › nanomaterials-1281220-supplementary.pdf]

Supplementary Materials for manuscript "MXene Core-Shell Nanosheets: Facile Synthesis, Optical Properties and Vesatile Photonics Applications"

Yunjia Wang, Shunxiang Liu, Feng Zhu, Yiyu Gan and Wen Qiao *

Table S1. Output performance comparison of mode-locked fiber lasers using various 2D materials SAs at a wavelength of 1.55 um.

Materials	SA	Modulation Depth (%)	Output Power (mW)	Pump Power (mW)	Pulse Width (fs)	3-dB Spectral Width (nm)	Radio Frequency (dB)	Ref.
GR	Graphene	66.5	2	70	756	NA	65	[1]
	GR oxide	1.4	0.83	34.5	613	4.2	70	[2]
BP	BP	9	2	30	940	3.39	50	[3]
	BPQDs	5	NA	75	1007	3.4	50	[4]
TIs	Bi_2Se_3	15.7	0.8	55	600	4.63	65	[5]
	Sb_2Te_3	NA	0.5	45	1800	1.8	60	[6]
TMDs	WS_2	5.1	1.8	85	21100	14.5	NA	[7]
	MoS_2	4.3	1.78	22	710	4	60	[8]
MXenes	$Ti_3C_2T_x$	29.3	NA	50	NA	NA	NA	[9]
	$Ti_3C_2T_x$	NA	NA	263	530	5.04	NA	[10]
	$Ti_3C_2T_x$	0.96	NA	146	1280	2.71	71	[11]
	$Ti_3C_2T_x$	11.3	0.283	118.5	597	5.21	55.2	[12]
	$Ti_3C_2T_x$	4.75	1.4	70	114	25.6	65	[13]
	$Ti_3C_2T_x$	20	3	238	159	22.2	62	[14]
	$Ti_3C_2T_x$	41	NA	60	946	3.1	70.7	[15]
	$Ti_3C_2T_x$	NA	3.48	400	800	3.75	65	[16]
	Ti_3CNTx	1.7	0.05	22	660	5	60	[17]
	α-Mo_2C	4.29	NA	127.1	2790	1.66	45	[18]
	α-Mo_2C	10.45	NA	100	313	13.75	79	[19]
	Ti_2CT_x	NA	2.91	102	265	11.1	47	[20]
	Ti_2CT_x	15.7	NA	65	5300	3.4	62	[21]

**Ti_2CT_x@Au**	Ti_2CT_x@Au	6.6	0.491	20	670	3.2	62	This work

Table S2. Progress of SFFLs based on LCLM with 2D material

SA	Gain Medium	Linewidth [kHz]	SNR [dB]	Power [mW]	Power Fluctuation [%]	Reference
Graphene	YDF	NA	~60	16	NA	[22]
MoS_2	YDF	5.89	~60	15.3	<±2.7	[23]
Ni-MOFs	EDF	3.2	>52	1.07	<1.3	[24]
MXene QDs	EDF	5	54	7.6	<0.75	[25]
Ti_2C	EDF	1.3	>38	1.44/5.34	<1.8/<7.1	[26]
Ti_2C@Au	EDF	1	48.77	1.18/11.16	0.76/1.34	This Work

References

1. Bao, Q.L.; Zhang, H.; Wang, Y.; Ni, Z.H.; Yan, Y.L.; Shen, Z.X.; Loh K.P.; Tang, D.Y. Atomic-Layer Graphene as a Saturable Absorber for Ultrafast Pulsed Lasers. *Adv. Funct. Mater.* **2009**,19, 3077-3083.

2. Boguslawski, J.; Sotor, J.; Sobon, G.; Kozinski, R.; Librant, K.; Aksienionek,M.; Lipinska, L.;Abramski, K.M. Graphene oxide paper as a saturable absorber for Er- and Tm-doped fiber lasers. *Photonics Res.* **2015**, *3*, 119-124.

3. Luo, Z.C.; Liu, M.; Guo, Z.N.; Jiang, X.F.; Luo, A.P.; Zhao, C.J.; Yu, X.F.; Xu, W.C.; Zhang, H. Microfiber-based few-layer black phosphorus saturable absorber for ultra-fast fiber laser. *Opt. Express*. **2015**, *23*, 20030-20039.

4. Xu, Y.; Wang, W.; Ge, Y.; Guo, H.; Zhang, X.; Chen, S.; Deng, Y.; Lu, Z.; Zhang, H. Stabilization of black phosphorous quantum dots in PMMA nanofiber film and broadband nonlinear optics and ultrafast photonics application. *Adv. Funct. Mater.* **2017**, *27*, 1702437-1702443.

5. Lee, J.S.; Koo, J.; Jhon, Y.M.; Lee J.H. A femtosecond pulse erbium fiber laser incorporating a saturable absorber based on bulk-structured Bi_2Te_3 topological insulator. *Opt. Express* **2014**, *22*, 6165-6173.

6. Sotor, J.; Sobon, G.; Macherzynski, W.; Paletko, P.; Grodecki, K.; Abramski, K.M. Mode-locking in Er-doped fiber laser based on mechanically exfoliated Sb_2Te_3 saturable absorber. *Optical Materials Express* **2014**, *4*, 1-6.

7. Mao, D.; Zhang, S.L.; Wang. Y.D.; Gan, X.T.; Zhang, W.D.; Mei, T.; Wang, Y.G.; Wang, Y.S.; Zeng, H.B.; Zhao, J.L. WS_2 saturable absorber for dissipative soliton mode locking at 1.06 and 1.55 um. *Opt. Express* **2015**, *23*, 27509-27519.

8. Liu, H.; Luo, A.P.; Wang, F.Z.; Tang, R.; Zhang, H. Femtosecond pulse erbium-doped fiber laser by a few-layer MoS_2 saturable absorber. *Opt. Lett.* **2014**, *39*, 4591-4594.

9. Wang, Z.H.; Li, H.B.; Luo, M.L.; Chen, T.H.; Xia, X.F.; Chen, H.L.; Ma, C.Y.; Guo, J.; He, Z.W.; Song, Y.F.; Liu, J.; Jiang, X.T.; Zhang, H. MXene photonic devices for near-Infrared to mid-Infrared ultrashort pulse generation. *ACS Appl. Nano Mater.* **2020**, *3*, 3513-3522.

10. Je, K.J.; Min, K.C.; Bae, L.S.; Kwanil, L. *Novel sturable absorber based on 2D nanomaterial, MXene solution* **2019**.

11. Feng, J.; Li, X.; Feng, T.; Wang, Y.; Liu, J.; Zhang, H. Harmonic Mode-Locked Er-Doped Fiber Laser by Evanescent Field-Based MXene Ti_3C_2Tx (T= F, O, or OH) Saturable Absorber *Ann. Phys. (Germany)* **2020**,532, 1900437-1900443.

12. Wu, Q.; Jin, X.; Chen, S.; Jiang, X.; Hu, Y.; Jiang, Q.; Wu, L.; Li, J.; Zheng, Z.; Zhang, M.; Zhang, H. MXene-based saturable absorber for femtosecond mode-locked fiber lasers *Opt. Express*. **2019**, *27*, 10159-10170.

13. Jiang, X.; Li, W.; Hai, T.; Yue, R.; Chen, Z.; Lao, C.; Ge, Y.; Xie, G.; Wen, Q.; Zhang, H. Inkjet-printed MXene micro-scale devices for integrated broadband ultrafast photonics *npj 2D Mater. Appl.* **2019**, *3*, 1-9.

14. Jiang, X.; Liu, S.; Liang, W.; Luo, S.; He, Z.; Ge, Y.; Wang, H.; Cao, R.; Zhang, F.; Wen, Q. Broadband nonlinear photonics in few-layer MXene Ti_3C_2Tx (T= F, O, or OH). *Laser & Photonics Reviews* **2018**, *12*, 1700221-1700229.

15. Li, J.; Zhang, Z.; Du, L.; Miao, L.; Yi, J.; Huang, B.; Zou, Y.; Zhao, C.; Wen, S. Highly stable femtosecond pulse generation from a MXene Ti_3C_2Tx (T = F, O, or OH) mode-locked fiber laser. *Photonics Res.* **2019**,*7*, 260-264.

16. Je, K.J.; Hyerim, K.; Min, K.C.; Ha, L.J.; Bae, L.S.; Kwanil, L. Femtosecond Mode-locked Fiber Laser Using an Etched Optical Fiber Immersed in Liquid Mxene as a Saturable Absorber 23rd Opto-Electronics and Communications Conference (OECC), **2018**, *2*.

17. Jhon, Y.I.; Koo, J.; Anasori, B.; Seo, M.; Lee, J.H.; Gogotsi, Y.; Jhon, Y.M. Metallic MXene saturable absorber for femtosecond mode-locked lasers. *Adv. Mater*. **2017**, *29*, 1702496-1702502.

18. Tuo, M.; Xu, C.; Mu, H.; Bao, X.; Wang, Y.; Xiao, S.; Ma, W.; Li, L.; Tang, D.; Zhang, H.; Premaratne, M.; Sun, B.; Cheng, H.M.; Li, S.; Ren, W.; Bao, Q. Ultrathin 2D Transition Metal Carbides for Ultrafast Pulsed Fiber Lasers *Acs Photonic.* **2018**, *5*, 1808-1816.

19. Liu, S.; Wang, Y.; Lv, R.; Wang, J.; Wang, H.; Wang, Y.; Duan, L. 2D molybdenum carbide (Mo_2C)/fluorine mica (FM) saturable absorber for passively mode-locked erbium-doped all-fiber laser. *Nanophotonics* **2020**, 9, 2523-2530.

20. Shi, Y.H.; Xu, N.; Wen, Q. Ti_2CTx (T=O, OH or F) Nanosheets as New Broadband Saturable Absorber for Ultrafast Photonics. *J. Lightwave Technol.* **2020**, *38*, 1975-1980.

21. Yi, J.; Du, L.; Li, J.; Yang, L.; Hu, L.; Huang, S.; Dong, Y.; Miao, L.; Wen, S.; Mochalin, V.N. Unleashing the potential of Ti_2CT x MXene as a pulse modulator for mid-infrared fiber lasers. *2D Mater.* **2019**, *6*, 045038-045043.

22. Deng, J.; Chen, H.; Lu, B.; Yin, M.; Li, D.; Jiang, M.; Bai, J. Single frequency Yb-doped fiber laser based on graphene loop mirror filter. *Journal of Optics* **2015**,*17*, 025802-025809.

23. Lu, B.; Yuan, L.; Qi, X.; Hou, L.; Sun, B.; Fu, P.; Bai, J. MoS_2 saturable absorber for single frequency oscillation of highly Yb-doped fiber laser. *Chinese Optics Letters* **2016**, *14*, 071404-071411.

24. Sun, Z.H; Jiang, X.J.; Wen, Q.; Li, W.J; Zhang, H. Single frequency fiber laser based on an ultrathin metal–organic framework *J. Mater. Chem. C* **2019**, 7, 4662-4666

25. Xu, N.; Li, H.; Gan, Y.; Chen, H.; Li, W.; Zhang, F.; Jiang, X.; Shi, Y.; Liu, J.; Wen, Q.; Zhang, H. Zero-Dimensional MXene-Based Optical Devices for Ultrafast and Ultranarrow Photonics Applications. *Adv. Sci.* **2020**,*7*, 2002209-2002216.

26. Gan, Y.; Zhu, F.; Shi, Y.; Wen, Q. Single frequency fiber laser base on MXene with kHz linewidth. *J. Mater. Chem. C* **2021**,*9*, 2276-2281.
